# Isolation of a bacterial strain from the gut of the fish, *Systomus sarana*, identification of the isolated strain, optimized production of its protease, the enzyme purification, and partial structural characterization

**DOI:** 10.1186/s43141-022-00299-3

**Published:** 2022-02-10

**Authors:** Arul Dhayalan, Balasubramanian Velramar, Balasubramani Govindasamy, Karthik Raja Ramalingam, Aiswarya Dilipkumar, Perumal Pachiappan

**Affiliations:** 1 Department of Biotechnology, School of Biosciences, Periyar University, Salem, 636011 Tamil Nadu India; 2 ICAR- National Dairy Research Institute, SRS, Adugodi, Bengaluru, 560030 Karnataka India; 3 Amity Institute of Biotechnology, Amity University, Raipur, 493225 Chhattisgarh India; 4 ICAR- Central Institute of Brackishwater Aquaculture, Chennai, 600028 Tamil Nadu India; 5 Department of Microbiology, Alagappa University, Karaikudi, 630003 Tamil Nadu India; 6 1/145, New Mariyaman Kovil Street, Bominayakanpatti post, Pagalpatti, Salem, 636304 Tamil Nadu India; 7 Department of Marine Science, School of Marine Sciences, Bharathidasan University, Tiruchirappalli, 620024 Tamil Nadu India

**Keywords:** *Bacillus thuringiensis*, Enzyme assay, Peptide fingerprinting, Homology modeling, Response surface methodology

## Abstract

**Background:**

The present study focuses on the isolation of *Bacillus thuringiensis* bacterium from the gut of fresh water fish, *Systomus sarana*, the innovative optimization of culture parameters to produce maximum protease enzyme, by the isolated bacterium, and the elucidation of peptide profile of the protease. And the experimental data and results were authenticated through the response surface method (RSM) and Box-Behnken design (BBD) model.

**Results:**

During the RSM optimization, the interaction of the highest concentrations (%) of 2.2 maltose, 2.2 beef extract, and 7.0 pH, at 37 °C incubation, yielded a maximum protease enzyme of 245 U/ml by the fish gut-isolated, *B. thuringiensis*. The spectral analysis of the obtained enzyme revealed the presence of major functional groups at the range of 610–3852 cm^−1^ *viz.*, alkynes (–C≡C–H: C–H stretch), misc (P-H phosphine sharp), α, β-unsaturated aldehydes, and through PAGE analysis, its molecular weight was determined as 27 kDa. The enzyme’s MALDI-TOF/MS analysis revealed the presence of 15 peptides from which the R.YHTVCDPR.L peptide has been found to be a major one.

**Conclusions:**

The fish gut-isolated bacterium, *B. thuringiensis*, SS4 exhibited the potential for high protease production under the innovatively optimized culture conditions, and the obtained result provides scope for applications in food and pharmaceutical industries.

**Supplementary Information:**

The online version contains supplementary material available at 10.1186/s43141-022-00299-3.

## Background

Microorganisms play a vital role in all ecosystems by driving the major biogeochemical cycles, and thus they contribute to almost half of the global primary productivity [1]. In view of the significant role of microbial enzymes in biochemical processes, the microbes are considered to be an integral part of biotechnological research. Traditionally produced industrial enzymes are used as biocatalysts with broader applications in various industrial sectors since they are cheaper and eco-friendly, and they can be also used as a substitute for synthetic chemicals during the processing of complex substrates [2]. The gastrointestinal (GI) tract of fish is being colonized by a number of beneficial bacteria called “probiotics.” The term probiotic referred to “for life,” as opposed to the term antibiotic, which means “against life.” Fish bodies have a symbiotic relationship with these probiotics. And such microbes help to digest the food, kill the harmful microorganisms, and keep the proper- body functioning of the fishes [3].

Proteases are a group of proteolytic enzymes that catalyze and breakdown the proteins through hydrolysis of peptide bonds between the amino acids of the polypeptide chains. Proteases have been successfully produced by researchers from different microbial sources. Microbes account for a two-thirds share of commercial protease production around the world [4]. Since the advent of enzymology, microbial proteolytic proteases have been the most widely studied enzyme. These enzymes have gained prominence not only due to their vital role in metabolic activities but also due to their immense utilization in industries [5]. The industrial use of such enzymes accounts for nearly 60% of the total enzyme market [6–8]. Sources of proteases include all forms of life *viz.*, plants, animals, and microorganisms. Based on their acid-base behavior, proteases are classified into three groups: acid, neutral, and alkaline proteases. Acid proteases performed best at the pH range of 2.0–5.0 and are mostly produced by fungi. Proteases that require around 7 pH are called neutral proteases that are mainly of plant origin, whereas the proteases showing maximum activity at the pH range of 8 and above are the alkaline ones from microorganisms [9].

Although the protease-enzyme is industrially important, its bulk production involves high costs. Moreover, the presently available microbial culture medium composition shows only moderate influence on enzyme yield, and therefore, formulating a proper fermentation medium is important. The culture medium optimization is a process where components of medium and culture parameters are suitably changed/optimized so as to get better growth/high productivity of the organisms [10]. Therefore, more studies are needed on the optimization of fermentation medium and process conditions to maximize the enzyme production. Some investigations were already carried out in submerged fermentation, in relation to factors like different concentrations of carbon and nitrogen [7].

Several statistical approaches like Box-Behnken design (BBD) and response surface methodology (RSM) have provided scope for the optimization for maximum enzyme production. The Box-Behnken design has been used for the screening of the main factors from a large number of variables, and this information can be retained in further optimization [11]. The present focus is therefore towards the optimization of different components of the medium by innovative statistical methods. Optimization by conventional methods (one factor at a time) is by varying one parameter at a time while fixing other parameters constantly. This method helps to assess the importance of that parameter on the enzyme production [12].

The objective of this work was to isolate the bacterium from the freshwater barb fish gut, molecular identification of the isolated bacterium, and to optimize the growth parameters for enhancing the protease yield of the *Bacillus thuringiensis* bacterium in potential casein substrate, by adopting innovative statistical tools.

## Methods

### Isolation of protease producing bacterium

Samples of cyprinid fish (*Systomus saranus*) were collected from the wild, Cauvery River at Stanley Reservoir, Tamil Nadu, India (21°43.232′N, 87°48.884′E), by using drag net, and the pH 7.8–8.4, water temperature, 27.3–30.1 °C, and salinity 16–18 ppt were recorded then there. The homogenates of the fish gut tissue were serially diluted up to 10^−5^ in sterilized saline, and 100 μl from each dilution was spread on tryptic soy agar (TSA, 30 g/L) plates and then incubated aerobically. After enumerating the microbial colonies by plate counting method, pure cultures were raised by using the morphologically different colonies.

### Screening of enzyme and enzyme index (EI)

The protease activity of isolates was screened by a tyrosine hydrolysis test on skim milk agar (SMA) (0.1% peptone, 0.5% NaCl, 2% skim milk, and 2% agar). After streaking of isolates and incubation and 1% iodine flooding, the appearance of clear zones confirms the activity [13]. The EI was expressed by the relationship between the average diameter (dm) of the degradation halo and the colony growth (EI = dm of hydrolysis zone/dm of colony) [14].

### Protease enzyme assay

Each isolate was inoculated in to the medium (0.1% peptone, 0.5% glucose, 0.05% NaCl (w/v), and 0.01% MgSO4.7H_2_O) and put in shaking incubator (150 rpm, 48h). The culture-pellet was centrifuged (10,000 rpm; 150 min at 4 °C), then added to it the 500 μL of 1% casein in 50 mM phosphate buffer (pH 7) and 200 μl of cell-free supernatant, then incubated in a water bath (40 °C, 20 min), and the reaction was terminated with the addition of 1mL of 10% TCA, subsequently kept at room temperature (15 min). The unreacted casein was separated by centrifuging the mixture, finally the supernatant was added with Na2Co3 (2.5 m ML) and Folin-Ciocalteu phenol and incubated in dark room (30 min). The OD was taken at 660 nm, using the standard [15]. One unit of protease is defined as the amount of enzyme that releases 1 μg/ml/min of tyrosine.

### “DNA extraction,” “PCR amplification,” “Agarose gel electrophoresis,” and “sequence analysis”

Bacterial genomic DNA (gDNA) extraction was done according to the method of Sambrook et al. [16] (with slight modification). A volume of 1.5 ml culture broth was centrifuged at 8000 rpm for 10 min. And the collected pellet was suspended in 450 μL of TE buffer (vortex mixer). To that pellet, 5 μl of lysozyme and 50 μl of 10% sodium dodecyl sulfate (SDS) were added and incubated at room temperature for 1 h. After incubation, an equal volume of phenol:chloroform (1:1) was added, mixed well, and centrifuged at 8000 rpm for 10 min. Then, the aqueous phase was transferred to a fresh tube without disturbing the bottom layer. To the aqueous solution, 50 μL of 3M NaCH_3_ (sodium acetate) and 300 μL of isopropanol were added to precipitate the DNA. Then the mixture was centrifuged at 10,000 rpm for 10 min and the supernatant was discarded. The collected pellet was washed with 70% ethanol and centrifuged at 5000 rpm for 1 min. Ethanol was discarded and evaporated without losing DNA. Then the DNA was dissolved with 50 μL of TE buffer and the DNA samples were stored at – 20 °C. The partial 16S rRNA gene sequence was amplified using universal primers: 27F (5′ AGAGTTTGATCMTGGCTCAG 3′) and 1525R (5′AAG GAG GTG ATCCAGCCGCA 3′). The reaction mixture (25 μL) for PCR amplification was prepared with de-ionized water (7.3 μL), 10X Taq buffer (2.5 μL), forward primer (1 μM) 1.0 μL, reverse primer (1 μM) 1.0 μL, dNTPs (10 mM) 2.0 μL, Taq polymerase (3 U/μL) 0.2 μL, and DNA template 1.0 μL. The program was conducted using 35 cycles of initial denaturation at 94 °C for 5 min, denaturation at 94 °C for 60 s, annealing at 55 °C for 45 s, and elongation at 72 °C for 1 min 30 s with a final extension at 72 °C for 7 min (Verti™ Thermal Cyclers, Applied Biosystems). The PCR products were analyzed on 1% agarose gel. Agarose was dissolved completely in the 1X TBE (Tris-Boric Acid-EDTA) buffer and heated in a microwave oven for 5 min. Before solidification at 45 °C, 3 μL of ethidium bromide (EtBr) was added into the gel solution for visualization of bands. Five microliters of DNA sample was mixed with 2 μL of 2X loading dye and loaded into the wells. Amplified products were visualized by placing the gel in a UV documentation system (Bio-Rad, Italy). Sequence data was analyzed through the NCBI database (www.ncbi.nlm.nih.gov) by using the BLAST program. The unknown sequence was compared to all of the sequences already available in the database so as to assess the DNA similarities. The obtained nucleotide sequence was deposited in GenBank. Multiple sequence alignment and molecular phylogeny were performed using MEGA7 software [17]. Phylogenetic analysis was performed using the neighbor-joining method.

### Response surface methodology (RSM)

A 5-factor Box-Behnken design consisting of 46 investigational runs with 3 replications at the central point (Table 1) was used to optimize the independent variables, i.e., pH, temperature, carbon sources, nitrogen sources, and incubation day. The modeling and numerical analysis were performed using design expert, version 8.0.4.1 software (Stat-Ease Inc. Minneapolis). The quality of fit of the second-order model equation was expressed by the coefficient *R*^2^, and its statistical significance was determined by an *F* test. The data were interpreted to obtain the response surface in the form of contours and 3D descriptions by viewing the interaction of the factors.

**Table 1:** Analysis of variance (ANOVA) for response surface quadratic model (Box-Behnken)

Source	Sum of squares	df	Mean square	***F*** value	***p*** value Prob > ***F***	
Model	93,778.34	20	4688.917	8.9531	< 0.0001	Significant
A- pH	23.5225	1	23.5225	0.0449	0.8339	
B- Temperature	44.65581	1	44.65581	0.0852	0.7727	
C- Maltose	2872.96	1	2872.96	5.4857	0.0274	
D- Beef extract	887.891	1	887.891	1.6953	0.2048	
E- Incubation time	14.5924	1	14.5924	0.0278	0.8688	
AB	4160.25	1	4160.25	7.9437	0.0093	
AC	36.6025	1	36.6025	0.0698	0.7937	
AD	400	1	400	0.7637	0.3905	
AE	2545.203	1	2545.203	4.8599	0.0369	
BC	256	1	256	0.4888	0.4909	
BD	540.5625	1	540.5625	1.0321	0.3194	
BE	6.838225	1	6.838225	0.0130	0.9099	
CD	490.6225	1	490.6225	0.9368	0.3424	
CE	2116	1	2116	4.0403	0.0553	
DE	204.633	1	204.633	0.3907	0.5376	
A^2^	27,991.69	1	27,991.69	53.448	< 0.0001	
B^2^	24,876.63	1	24,876.63	47.500	< 0.0001	
C^2^	34,419.42	1	34,419.42	65.721	< 0.0001	
D^2^	37,710.85	1	37,710.85	72.006	< 0.0001	
E^2^	38,409.17	1	38,409.17	73.339	< 0.0001	
Residual	13092.88	25	523.715			
Lack of fit	13,092.88	20	654.6438	1.8657		Not significant
Pure error	0	5	0			
Cor total	106,871.2	45				

SD (22.885); mean (138.948); C.V % (16.470); PRESS (52371.504); *R*^2^ (0.907); adjusted *R*^2^ (0.779); predicate *R*^2^ (0.510); Adeq precision (10.644)

### Effect of culture

#### Parameters on protease enzyme production

The effective strain SS5 was inoculated into the production medium with the following parameters *viz*., pH 3–10, temperatures 10–45 °C, and incubation time 0–60 h in rotary shaking at 150 rpm. After incubation, the fermented content was centrifuged at 8000 rpm for 10 min at 4 °C, and the collected supernatants were used for protease enzyme assay in relation to the standard, D-galacturonic acid (μg/mL). The effect of six carbon sources (1%) (glucose, sucrose, maltose, galactose, lactose, and mannitol) and six nitrogen sources (1%) (yeast extract, peptone, (NH_4_)_2_SO_4,_ KNO_3,_ NaNO_3,_ and KH_2_PO_4_) on the production of protease were studied. The effective carbon and nitrogen sources were taken as 1 to 4% for inoculums and incubated at optimum conditions, after which protease assay (enzyme activity) was done [18].

#### Protein extraction, purification, and molecular mass determination

After incubation (150 rpm; 48h) of the 1×10^8^ microbial inoculum along with nutrient broth, the centrifuged culture material was added with acetone and the precipitated protein was obtained [19]. For gel filtration chromatographic assay, the sample was loaded into the glass column (packed with Sephadex G 100) and proteins were eluted using the Tris-HCL buffer (10mM, pH7.5). After that step, 1.5 ml of fractions was collected separately for the estimation of protein content and protease activity as shown in Table 2. The protein purification was determined in 12% SDS-PAGE [20], for which 20% of protein was loaded on 12% SDS-PAGE with standard molecule marker (GENEI, Bengaluru, India); then the gel was silver-stained. And the 2-D gel electrophoresis of the lyophilized protein sample was done at Sandor Life Science Pvt. Ltd., Hyderabad. In silico analysis of 2-D gel was performed on the basis of isoelectric point (pI) and molecular weight (MW) of separated proteins. The Expert Protein Analysis System (ExPASy), a SIB Bioinformatics Resource Portal, provides access to scientific databases and software tools in proteomics, genomics, phylogeny, and systems biology.

**Table 2:** Summary of purification factors of protease by *Bacillus thuringiensis* (SS5)

Purification steps	Protease activity(U)	Total protein (mg/ml)	Specific activity (U/mg)	Purification fold	Yield (%)
Optimized culture crude extract	483	213	2.3	1.0	100
Ammonium sulfate precipitation	421	106	3.9	1.58	87.16
Dialysis	368	43	8.8	2.21	76.19
Sephadex G-100	245	19	12.9	2.83	50.72

### High-performance liquid chromatography (HPLC) analysis

The extracellular protease enzyme protein- powder was diluted in 0.2 mM PBS and subjected to HPLC analysis. The samples were detected using an LC-20AD HPLC system (Shimadzu Chromatographic Instruments, Japan) equipped with a C_18_ reverse-phase column (particle size 5 μm and length 4.6 × 250 mm) and a SPD-20A UV/Vis detector at 272 nm absorbance with methanol [thin space (1/6-em)]: [thin space (1/6-em)] water (50[thin space (1/6-em)]: [thin space (1/6-em)]50) at a flow rate of 1 ml/min and head pressure of 300 kgf cm^−2^ [21].

### Fourier transform infrared spectroscopy (FT-IR) analysis

The functional groups of protease protein were analyzed using the ART model FT-IR Spectrophotometer. FT-IR spectra of 1% dry samples were scanned (Nicolet spectrophotometer) at the frequency range of 4000–400 cm^−1^ and at the resolution of 4 cm^−1^ using KBr discs [22].

### MALDI-TOF/MS analysis

The manually excised protein spots were digested overnight with trypsin at 37 °C. The 60% acetonitrile in 0.2% trifluoroacetic acid was used to extract the peptides from the gels, concentrated by vacuum drying, and desalted by using C18 reverse-phase micro-columns (OMIX Pipette tips, Varian). Micro-column-eluted peptide was added with 3 mL of matrix solution, and the mixture was subjected to direct analysis in a mass spectrometer sample plate. MALDI–TOF/MS spectrum was obtained by using MALDI–TOF/MS (Axima Performace, Kratos-Shimadzu, and Manchester, UK). The MALDI–TOF/MS data obtained from each protein digest was analyzed individually by using MASCOT software version 2.2 (Matrix Science, London, UK). Proteins were identified by NCBI database search with peptide m/z values using MASCOT search tool (URL http://www.matrixscience.com) for identification of tryptic maps [23].

### Homology modeling

Homology searches were performed with the NCBI BLAST server (http://www.ncbi.nlm.nih.gov/ BLAST/). Homology modeling was performed with SWISS-MODEL, a homology-modeling server, by following the protocol of Bordoli et al. [24]. The quality of predicted structural models was assessed through the stereochemical parameters of the Ramachandran plot.

### Statistical analysis

The obtained data were subjected to the statistical treatment of variance test (one-way ANOVA) by SPSS 20.1 software, along with Tukey’s tests, and the results at the level of *P*< 0.05 were considered to be significant. The data obtained from all the experiments were expressed as mean plus or minus standard error of three replicates.

## Results

### Fish- gut bacteria

Totally, 11 bacterial strains were isolated from the gut of the freshwater fish, *Systomus sarana*. The viable colonies were spotted from the TSA plates and the mono-populations were observed through a quadrant streak plate (Supplementary Figure S1A). The 11 isolated bacterial strains were named as SS1 to SS11, serially.

### Qualitative and quantitative assays

**Fig. 1:**
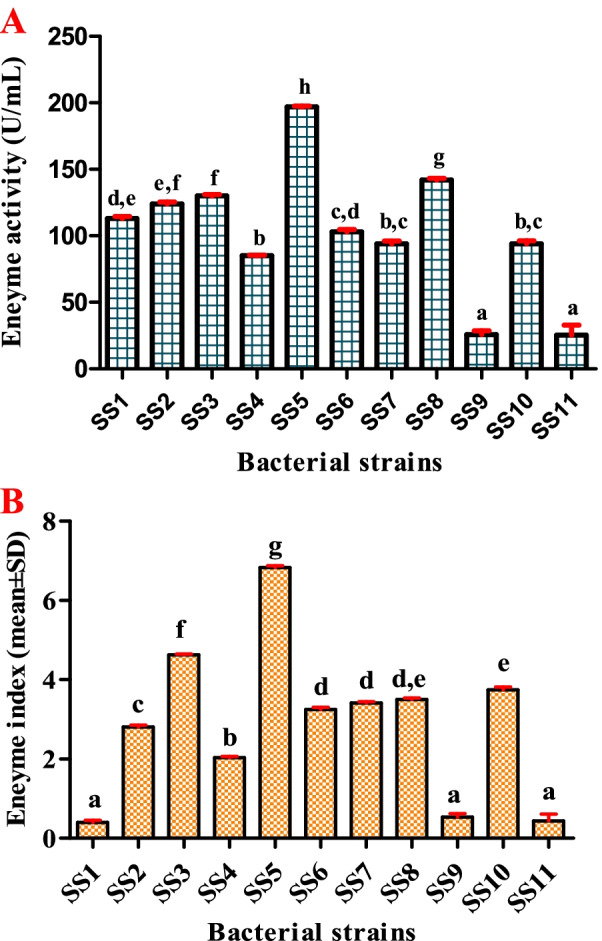
Quantitative analysis of protease enzymes producing bacterial strains (**A**); enzyme index (**B**)

All the isolated bacterial strains were screened for their nature of enzyme production. The cultured aerobic bacteria in the gut of collected fish revealed the production of protease enzymes. Only SS2, SS3, SS5, SS6, SS7, SS8, and SS10 were able to produce protease enzymes. From the initial screening of enzyme production, most of the bacterial strains have produced translucent zones around the colonies on the skim milk agar medium. Among them, the SS5 strain was found to produce the highest zone of enzyme production. The enzyme index of the SS5 strain revealed the highest zone of clearance as 6.83 mm (Fig. 1B). For the protease assay, cell-free supernatants of 11 bacterial strains were used for the quantitative assay of protease production. The SS5 bacterial strain exhibited maximum protease activity (197 U/mL) whereas the SS4 has produced only 85 U/mL amount of protease (Fig. 1A).

### Molecular identification of SS5 strain

The potential strain-SS5 was found to be a rod-shaped gram-positive bacterium (Supplementary Figure S1A). The PCR amplified fragment was found to comprise of approximately 1300 bp with 1 Kb DNA ladder (Supplementary Figure S1B). From the evolutionary analyses, a total of 1253 bp nucleotides were sequenced for 13 species. Plots of transitions and trans-versions against uncorrected genetic distance indicated an absence of nucleotide saturation in this gene. The optimal tree with the sum of branch length = 0.09432114 was observed from the constructed tree. The percentage of replicate trees in which the associated taxa clustered together in the bootstrap test (1000 replicates) is shown next to the branches. Based on the analysis, two major clades were obtained in the selected 13 species. One clade consists of a group of 2 species *viz.*, *Bacillus oryzaecorticis*and *Bacillus tequilensis*. The species *Bacillus thuringiensis* was resolved as *Bacillus* sp*.* with a high bootstrap value of BP = 56% (Supplementary Figure S1C).

### Optimization of SS5 strain culture conditions

**Fig. 2:**
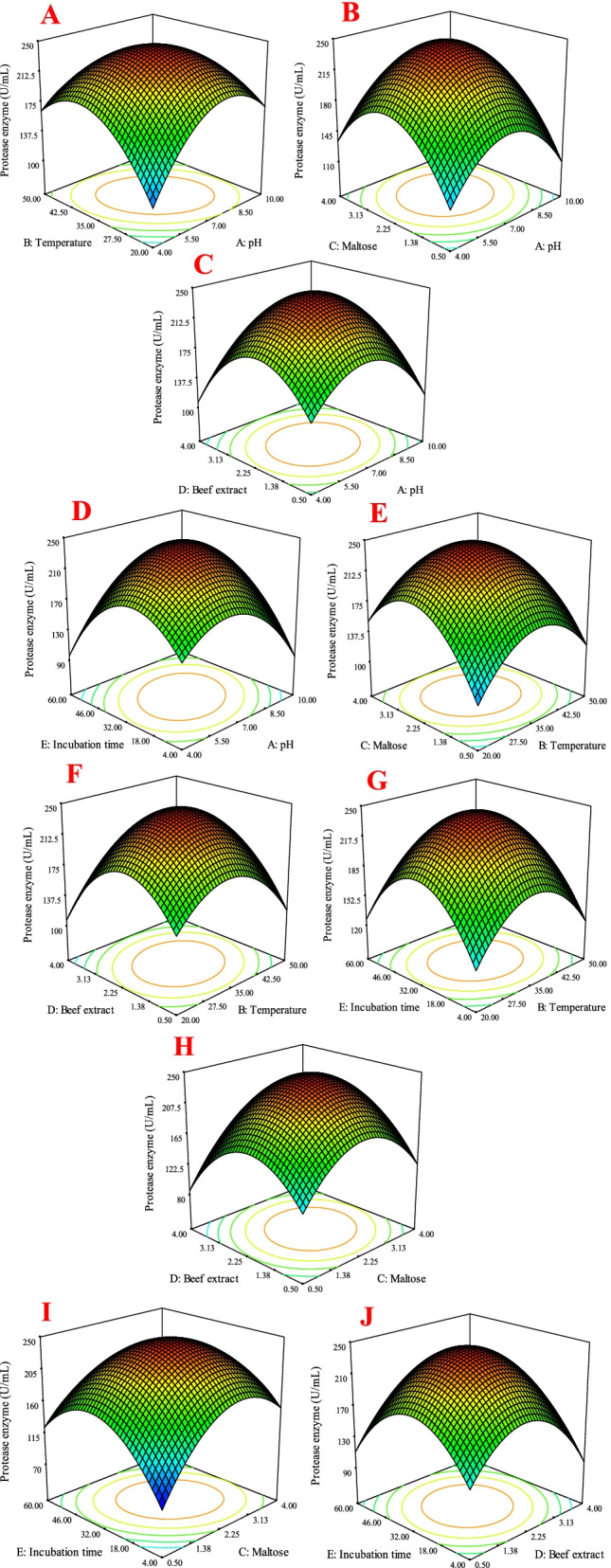
Five parameter analyses for the protease enzyme production culture condition of *Bacillus thuringiensis* by RSM

The culture conditions for protease enzyme production by SS5 strain were optimized by a total of 46 runs with 5 parameters through the BBD model. Table 1 shows the experimental values of about 95% significance of predicted values. The test variables were related by the following second-order polynomial equation. Through RSM method of analysis of protease enzyme production in relation to different parameters (under different variables), the enzyme production rate was 245.00 (+1.21*A-1.67*B+13.40*C-7.45*D-0.96*E-32.25*A*B+3.03*A*C+10.00*A*D+25.23*A*E-8.00*B*C+11.62*B*D-1.31*B*E+11.07*C*D-23.00*C*E+7.15*D*E-56.63*A2-53.39*B2-62.80*C2-65.73*D2-66.34*E2 ( Table 3 and Fig. 2).

**Table 3:** Composition of various experiments of the PBD for independent variables and response

Run	pH	Temperature	Maltose	Beef extract	Incubation time	Protease enzyme	
						Experimental value	Predicted value
1	7.00	35.00	2.25	0.50	4.00	130	128.48
2	10.00	35.00	0.50	2.25	32.00	101.5	110.35
3	7.00	35.00	2.25	2.25	32.00	244.9	244.18
4	4.00	35.00	2.25	2.25	4.00	135.8	146.99
5	7.00	35.00	4.00	0.50	32.00	106	126.24
6	10.00	50.00	2.25	2.25	32.00	99	102.27
7	7.00	35.00	4.00	2.25	4.00	145	153.21
8	7.00	50.00	2.25	2.25	4.00	138	125.86
9	10.00	35.00	2.25	0.50	32.00	99	121.29
10	7.00	20.00	2.25	2.25	60.00	109.23	127.29
11	4.00	35.00	2.25	2.25	60.00	105.9	94.63
12	10.00	35.00	4.00	2.25	32.00	165.6	143.20
13	7.00	35.00	2.25	2.25	32.00	243.5	244.18
14	7.00	20.00	2.25	2.25	4.00	118	126.59
15	4.00	20.00	2.25	2.25	32.00	84	103.18
16	7.00	35.00	2.25	2.25	32.00	243.5	244.18
17	10.00	35.00	2.25	2.25	60.00	178	147.51
18	4.00	35.00	0.50	2.25	32.00	84	113.98
19	4.00	35.00	2.25	4.00	32.00	137	103.97
20	7.00	35.00	2.25	4.00	60.00	102.5	111.67
21	7.00	35.00	2.25	4.00	4.00	106	99.28
22	7.00	20.00	2.25	0.50	32.00	175	146.62
23	7.00	50.00	2.25	4.00	32.00	106	128.38
24	4.00	35.00	4.00	2.25	32.00	136	134.73
25	7.00	35.00	2.25	2.25	32.00	245	244.18
26	10.00	20.00	2.25	2.25	32.00	167	170.11
27	7.00	20.00	4.00	2.25	32.00	154	151.88
28	7.00	35.00	2.25	0.50	60.00	97.89	112.27
29	10.00	35.00	2.25	4.00	32.00	103	126.39
30	7.00	35.00	4.00	4.00	32.00	110	133.49
31	7.00	50.00	0.50	2.25	32.00	142	121.74
32	7.00	35.00	2.25	2.25	32.00	244	244.18
33	7.00	20.00	2.25	4.00	32.00	136	108.47
34	7.00	35.00	0.50	0.50	32.00	136	121.59
35	4.00	50.00	2.25	2.25	32.00	145	164.34
36	7.00	35.00	0.50	4.00	32.00	95.7	84.54
37	7.00	20.00	0.50	2.25	32.00	100	109.08
38	7.00	35.00	2.25	2.25	32.00	244.2	244.18
39	7.00	50.00	4.00	2.25	32.00	164	132.54
40	7.00	50.00	2.25	0.50	32.00	98.5	120.03
41	7.00	35.00	0.50	2.25	60.00	127	124.50
42	10.00	35.00	2.25	2.25	4.00	107	98.97
43	4.00	35.00	2.25	0.50	32.00	173	138.87
44	7.00	50.00	2.25	2.25	60.00	124	121.34
45	7.00	35.00	4.00	2.25	60.00	100	105.30
46	7.00	35.00	0.50	2.25	4.00	80	80.41

The determination of coefficient (*R*^2^ = 0.907) was shown by ANOVA of the quadratic regression model, indicating that only 0.65% of the total variations was not explained by the model. The value of the adjusted determination of coefficient (*R*^2^ = 0.779) also confirmed that the model was highly significant. At the same time, a very low value of 16.470 of the coefficients of the variation (C.V.) clearly indicated a very high degree of precision and a good deal of reliability of the experimental values (Table 3).

### HPLC and FT-IR analytical results

**Fig. 3:**
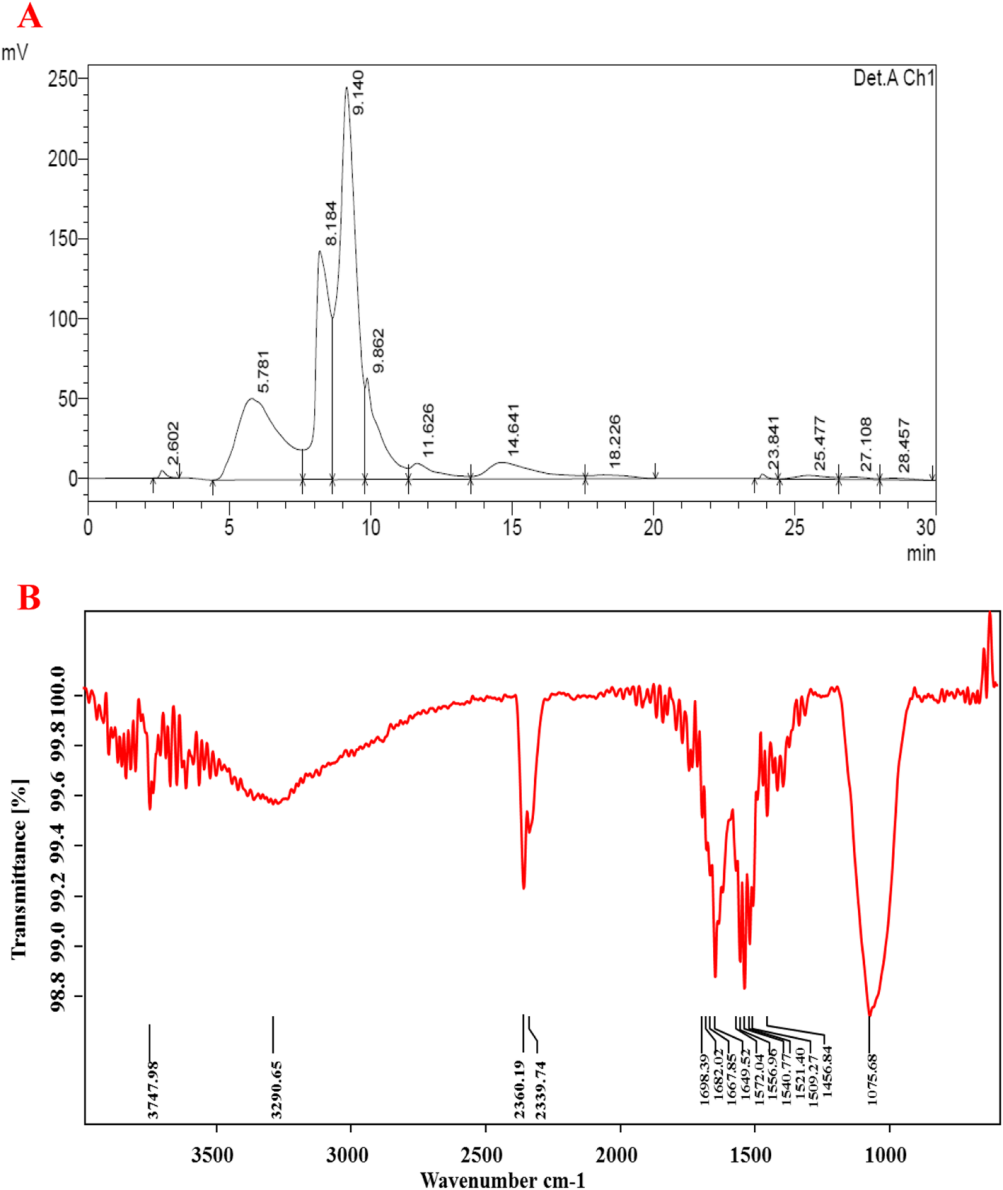
HPLC analysis of extracted proteins (**A**); FT-IR analysis of extracted protein for the protease enzyme-producing bacterium of *Bacillus thuringiensis* (**B**)

The HPLC analysis of the extracted protein revealed 12 peaks. Only one was a major peak at the retention time of 9.14 (45.63%) (Table S1). And the left behind nine minor peaks were found at the retention time of 2.60, 5.78, 8.18, 9.86, 11.62, 14.64, 18.22, 23.84, 25.47, 27.10, and 28.45 (Fig. 3A). Totally, 15 functional groups were identified through FT-IR analysis at the respective peaks of 3747, 3290, 2360, 2339, 1698, 1682, 1667, 1649, 1572, 1540, 1521, 1509, 1456, and 1075 cm^−1^. Based on the available literature and through the standard protocol, the identified functional classes were alkynes (–C≡C–H: C–H stretch), miscellaneous (Si-H silane), misc. (P-H phosphine sharp), α, β-unsaturated aldehydes, ketones (C=O stretch), alkenes (–C=C– stretch), alkenes (C=C stretch), 1° amines (N–H bend), carboxylic acid (C–O stretch), amides (NH out of plane), misc. (aromatic, nitro), misc. (N=O nitroso), alkanes (C–H bend), and ethers (C–O stretch) (Fig. 3B).

### Two-dimensional electrophoretic (2-DE) - results

**Fig. 4:**
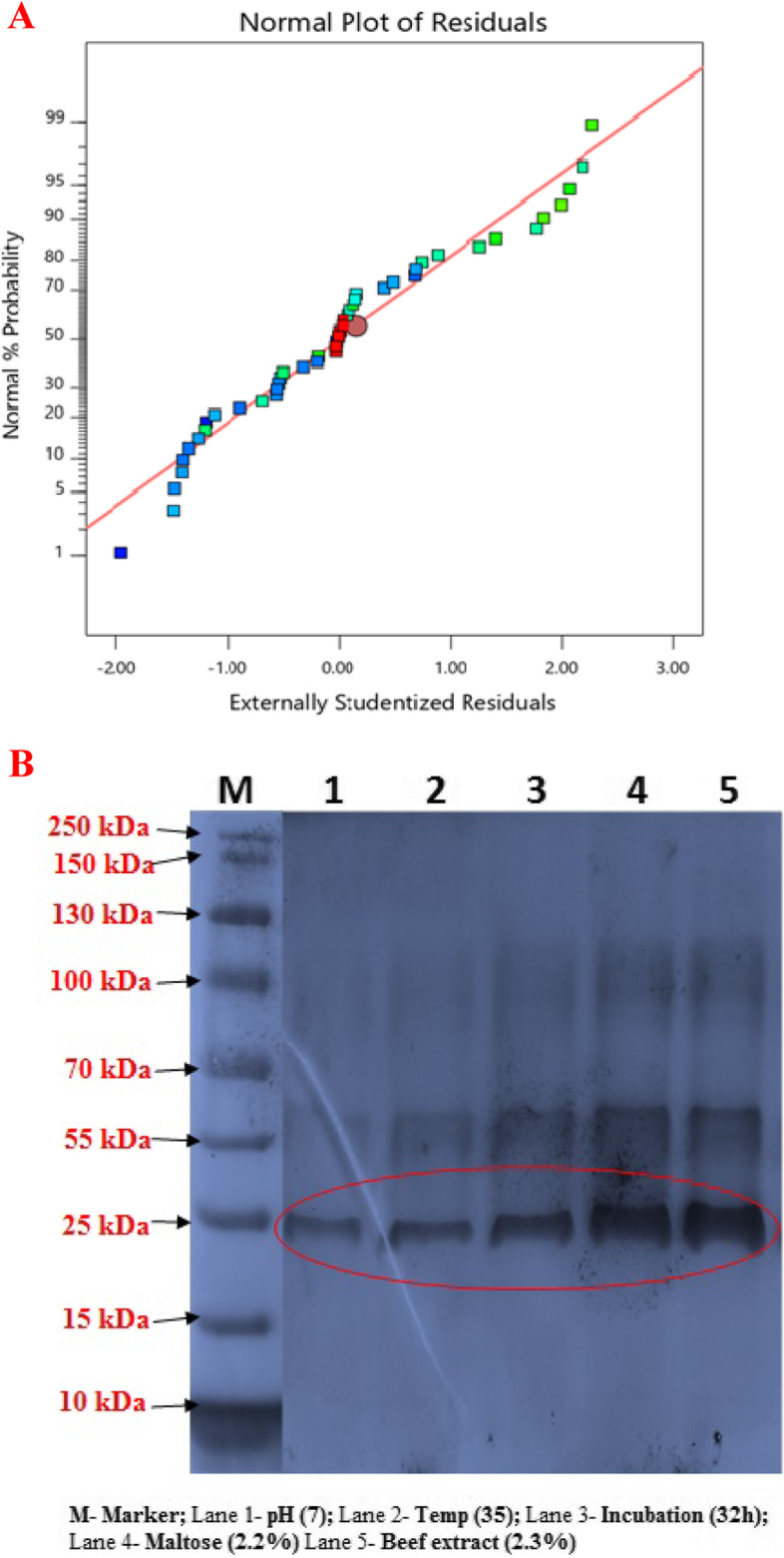
Normal probability plot of studentized residual (**A**); SDS-PAGE analyses by extracellular proteins (**B**)

The protein molecular weight was found to be 27 kDa as protease enzyme production (Fig. 4B). The 2-DE gel of proteins extracted from the bacterium *Bacillus thuringiensis* are shown in Supplementary Figure S2. The protein spots on the 2-D gels displayed isoelectric points (pI) at the pH range of 3–10 and the spots were concentrated between pH 6.5 and pH 7. Two-dimensional electrophoresis was performed on extracellular protease, when it achieved the highest extracellular protease enzyme activity (245 U/mL).

### Protein sequencing and homology modeling results

**Fig. 5:**
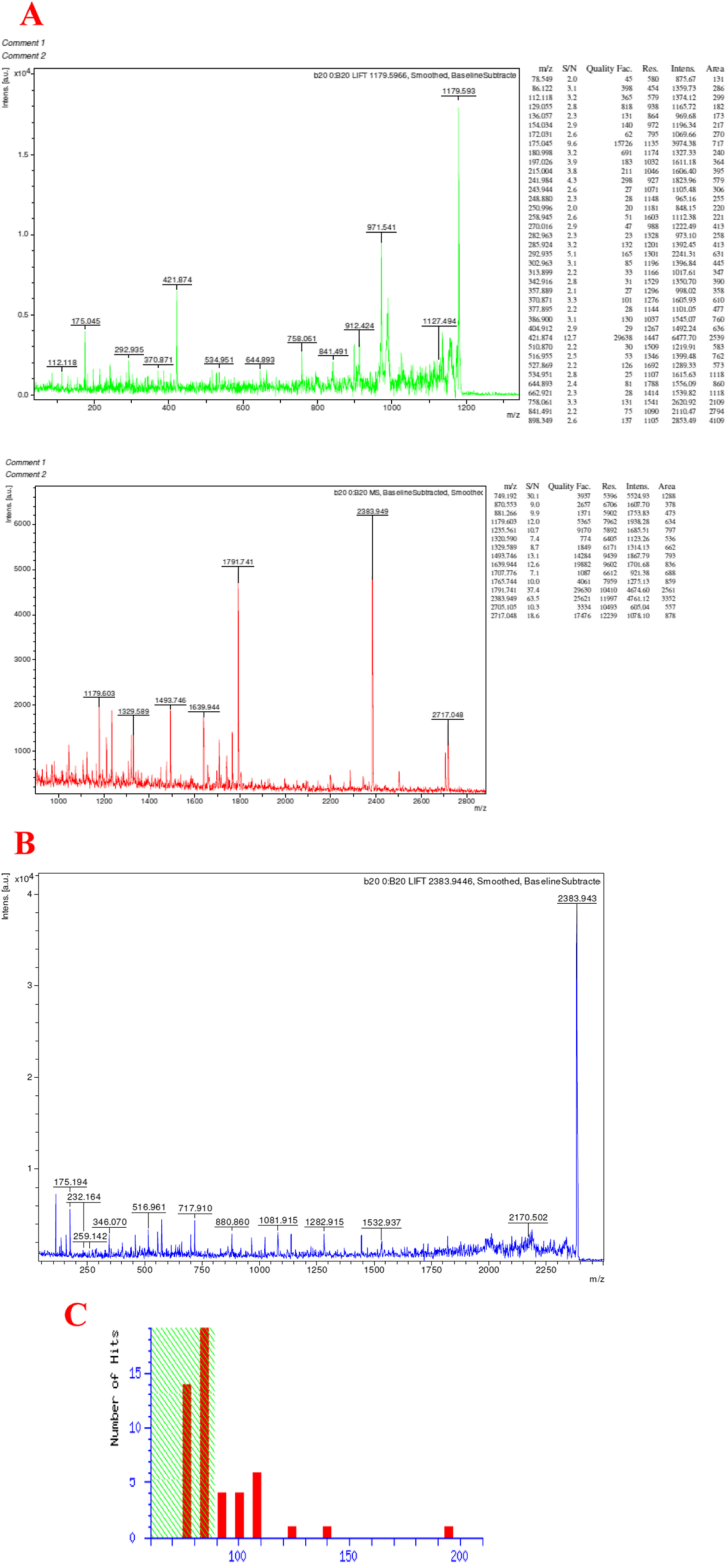
MALDI-TOF analysis: Intact mass determination (**A**); protein MS/MS 2383 spectrum (**B**); Mascot score histogram of the band corresponding to 23 kDa (**C**)

Partially purified protease was further subjected to MALDI–TOF analysis (Fig. 5A) and the molecular mass of purified protein was processed for the identification of specific proteins. High qualities of MS/MS spectra were obtained for 25 kDa protein (2383 spectra) (Fig. 5B). The monoisotopic masses of 25 kDa were pooled together and processed with MASCOT (Matrix Science) search; then it was found as protease (Fig. 5C).

**Fig. 6:**
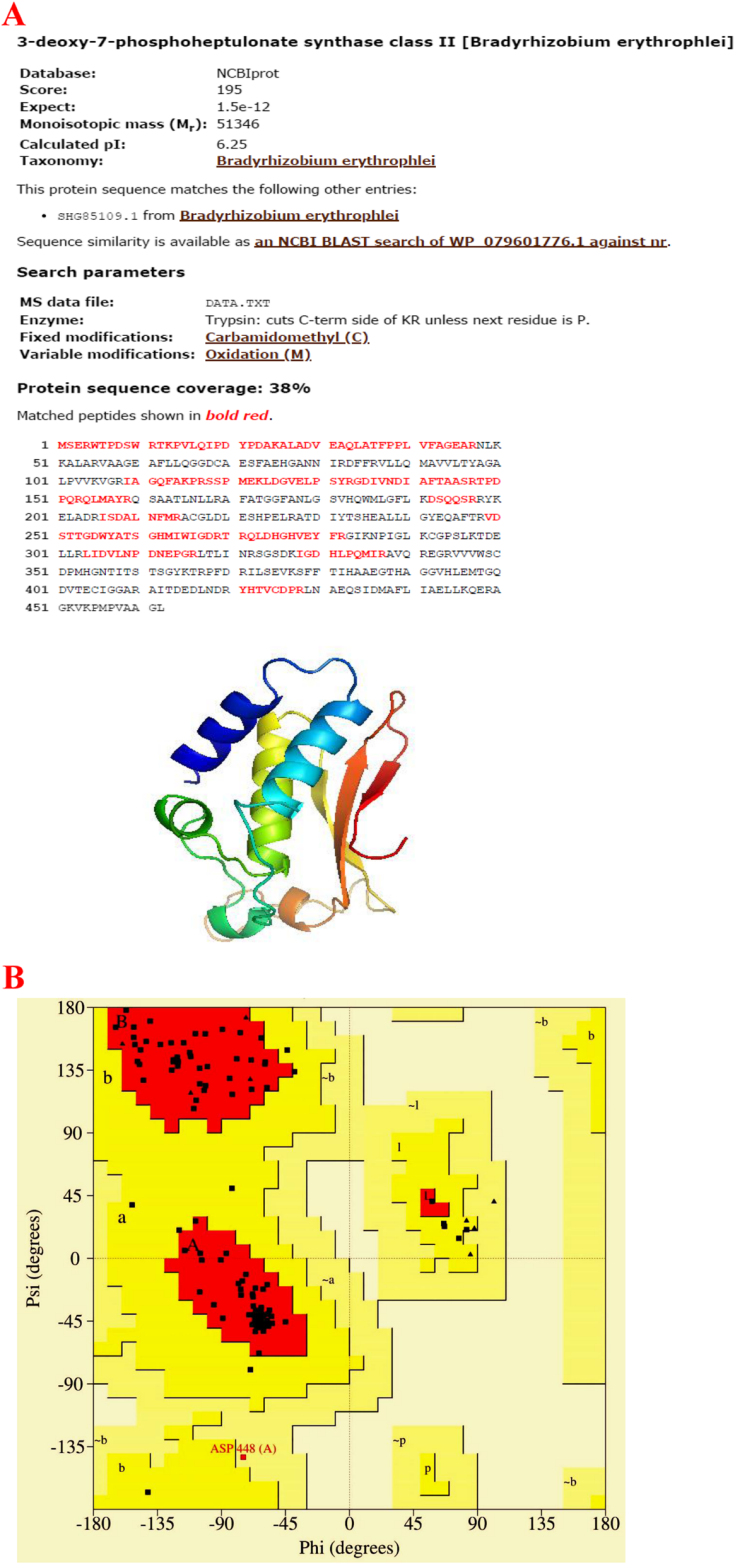
NCBI database for MS data of purified protease of *Bacillus thuringiensis* (**A**); Ramachandran plot of homology-modeled structure (**B**)

Differentially expressed 25 kDa protein was excised, in-gel tryptic digestion was done, and the protein analysis of enzyme sequence was found to consist of 462 residues (Fig. 6A) and also determined 15 peptides namely, -.MSERWTPDSWR.T, R.TKPVLQIPDYPDAK.A, K.ALADVEAQLATFPPLVFAGEAR.N, R.IAGQFAKPRSSPMEK.L, K.LDGVELPSYR.G, R.GDIVNDIAFTAASR.T, R.TPDPQRQLMAYR.Q, R.QLMAYR.Q, K.DSQQSR.R, R.ISDALNFMR.A, R.VDSTTGDWYATSGHMIWIGDR.T, R.TRQLDHGHVEYFR.G, R.LIDVLNPDNEPGR.L, K.IGDHLPQMIR.A, and R.YHTVCDPR.L followed as ppm 0.69, − 59.0, − 38.6, − 26.8, − 20.2, − 31.1, 9.46, − 11.3, − 36.4, − 32.8, − 42.0, − 14.1, − 68.2, − 23.1, and 87.1. Homology modeling of the predicted structure was plotted using a Ramachandran plot that revealed the distribution of φ and ψ angle in the model (Fig. 6B). According to this plot statistics, 90.7% of residues are located in the most favored region, 8.5% in the additionally allowed region, and 0.8% generally allowed region (Table 4), suggesting that its three-dimensional structure is likely to be similar to that of protease protein.

**Table 4:** Ramachandran plot analysis of proteins sequences

Ramachandran plot	Protease enzyme	Percentage
Residues in most favoured regions [A,B,L]	107	90.7%
Residues in additional allowed regions [a,b,l,p]	10	8.5%
Residues in generously allowed regions [~a,~b,~l,~p]	1	0.8%
Residues in disallowed regions	0	0.0%
Number of non-glycine and non-proline residues	118	100.0%
Number of end-residues (excl. Gly and Pro)	2	
Number of glycine residues (shown as triangles)	8	
Number of proline residues	5	
Total number of residues	133	

## Discussion

Of late, the production of microbial enzymes through the fermentation process has been improved with diverse renewable sources. Generally, for the production of industrial enzymes, the microbial cells are preferred from among the various groups of fungi, bacteria, and yeasts. Earlier, Sun et al. [25] reported that bacteria in fish gut have an important role in the digestion of food as well as in the immunity (of their hosts). Bacterial enzymes help digest the carbohydrates, proteins, and especially the substrate like cellulose which can be digested only by a few animals. According to Ray et al. [26], the extracellular enzyme-producing bacteria in fish gut exert positive effects on the digestive processes of the host. And this is evidenced through the results of the present screening of enzymes from the fish gut bacterium. Protease enzymes from the *Bacillus* species are the major industrial inputs in various important industrial areas *viz.*, leather processing, detergents, food, waste treatment, and peptide synthesis [27]. Previously, Blanco et al. [27] have reported that the protease enzyme production from *Bacillus* species has caught increased attention globally because of its important industrial applications in detergents, waste treatment, and in peptide synthesis. Though the proteases are produced by a variety of bacteria like *Pseudomonas aeruginosa*, *Flavobacterium*, *Clostridium*, *Achromobacter*, and thermo actinomyces besides *Streptomyces*, only the *Bacillus* spp. are considered to be a major source as they secrete a variety of soluble extracellular enzymes [28].

The present investigation well established that maximum enzyme production through SS5 could be achieved under the culture medium optimum of; pH 7.0, temperature 36.2v °C, maltose 2.2%, beef extract 2.3%, and incubation time 32 h. Earlier, Padmapriya et al. [29] have reported the highest protease enzyme activity at pH 7.0 by the species of *Bacillus* genus. Similarly, Govarthanan et al. [30] have optimized the culture conditions for *Bacillus* sp., and that yielded a maximum (920 U/mL) protease enzyme at the pH of 8.0 and temperature of 37 °C. The present results are consistent with some previous findings on the protease activity (821 U/mL) of *Bacillus* sp. Earlier, Bairagi et al. [31] observed that the optimum proteolytic activity by microbe was found between pH of 7.6 and 8.4. They have also suggested that physical parameters like pH and temperature largely influence the production rate and in relation to the type of species. For example, for fungal strains like *Fusarium* sp., the optimum pH and temperature have been reported to be 2.5 and 30 °C, respectively and the *Bacillus subtilis* bacterium exhibited good production at pH 7.0 and 35 °C temperature. Presently, the protease production by the bacterium *Bacillus thuringiensis* was achieved after 32 h incubation time, which is almost similar to the report of Asha and Palaniswamy [32], who have reported the incubation period at 48 h for the maximum protease enzyme production.

RSM is a collection of statistical tools that is useful for designing experiments, building models, evaluating the effects of different factors, and standardizing the optimal conditions of culture factors to get desirable responses [12]. Presently, some scattered spots along the trend-line satisfy the assumptions of the BBD model, which also indicates a normal distribution with accuracy and applicability of RSM (Fig. 4A). The RSM plots represent a comparison of the effects of all the factors at the midpoint (coded 0) in the design space (Fig. 2). A curvature with pH, temperature, incubation time, and maltose and beef extract concentration has shown the response to the level of maximum growth. The special features of the RSM tool are 3D response surface curve and point prediction from where we can determine the optimum value of the combination of the five parameters: pH (7.0), temperature (36.22), incubation time (32), maltose (2.20%), and beef extract (2.30%) that were responsible for the maximum protease enzyme activity (245 U/mL). The 3D response surface curve (Fig. 2A–J) determines the optimum condition of each component for maximum response. These plots were obtained from the pair-wise combination of independent factors while keeping another factor at its center point level.

Carbonaro and Nucara [33] have characterized the extracellular protease enzyme through FTIR analysis and obtained the characteristic absorption peaks that ranged between 1600 and 1700 cm^−1^, and from which they have found that the structure of the enzymatic hydrolysates contained C=C, C=C, C=N, C=N, −COOH, and −OH. In this connection, our present FTIR spectrum of protease revealed quite similar peaks that corresponded to the functional groups of α, β-unsaturated aldehydes from the *Bacillus thuringiensis* derived proteins*.* In an earlier study by Pelton and McLean [34], their IR spectrum revealed the amide II band at 1540–1550 cm^−1^ and a weaker shoulder at 1510–1525 cm^−1^. The antiparallel β-sheet structure of proteins with strong amide II bands between 1510 and 1530 cm^−1^; a parallel β-sheet structure was found at somewhat higher frequencies (1530–1550 cm^−1^).

During the present SDS analysis, the molecular weight (MW) of the protease was estimated to be ~25 kDa and 27 kDa, and these values closely tally with the previously reported 34 kDa value of serine protease from *B. pumilus* CBS [35]. Similarly, Uyar et al. [36] have also reported the extracellular protease enzyme value of 27 kDa in *B. cereus*. Likewise, Gessesse et al. [37] have purified a protease of 24 kDa from *Bacillus pseudofirmus*. Recently, Asker et al. [38] have purified the proteases from *Bacillus megaterium* that showed a specific activity of 317.23 U/mg proteins with a purification fold of 7.72, and they have determined its MW as 25 kDa.

Presently, the purified protein was quantitatively identified at 2383.943 m/z by the mass spectrum, which is considered to be the MW of 27 kDa. Recently, Chandrasekaran and Sathiyabama [39] have reported the MW of purified extracellular alkaline proteases as 42 kDa. They have found the sequence IKELATNGVVTNVK (378–391) segment of the alkaline serine protease through the MS/MS spectrum at 1485 m/z from the purified fraction. And they have confirmed the purified protein with an exact molecular mass of 43,074.11 Da. Earlier, alkaline proteases have been found to be reported, by researchers, from several fungal strains such as *Penicillium chrysogenum* FS010 (41 kDa), *Penicillium chrysogenum* Pg222 (35 kDa), and *Aspergillus clavatus* ES1 (30 kDa) [40–43]. Similar to some earlier findings, the presently purified protease enzymes showed the protein molecular weight that varied from 24 to 42 kDa. During our cost-benefit analysis for the industrial scale production of the protease enzyme, the cost of production of 2 l of enzyme was worked out to be Rs 2000 (approximately). The protease fermentation was scaled up in a 2-L conical flask with a titer of 7572 U/mL under optimized fermentation conditions. Further, it is of interest that presently the protein enzyme analysis revealed 462 residues and 15 peptides. During the plot statistics, 90.7% of residues have been found in the most favored region, 8.5% in the additionally allowed region, and 0.8% in generally allowed region (Table 4), revealing the three-dimensional structure of protease protein.

## Conclusion

The present study proved that the *B. thuringiensis,* isolated from the freshwater fish - gut, is an efficient protease producer under the optimized conditions of 7 pH, 36 °C temperature, and 2.2% substrate concentration at 32 h incubation that could yield 245 U/mL. To the best of our knowledge, this is the first report on the protease enzyme production by the Systomus sarana fish gut-isolated bacterium, *B. thuringiensis.* Based on the presently generated data, further investigation can be made for large-scale enzyme production and its industrial applications.

## Supplementary Information

**Additional file 1: Figure S1.** Protease enzyme producing bacterium SS5 culture strain, Gram negative bacteria (A); PCR amplified products (B); Phylogenetic tree derived from 16S rRNA sequences after comparing with strain SS5 using neighbor-joining method (C). **Figure S2.** 2D-Electrophrosesis of protein extract from *Bacillus thuringiensis*. **Table S1.** HPLC analysis of protease protein extract from *Bacillus thuringiensis* SS5.

## Data Availability

Materials used and data generated are available.

## Abbreviations

2-D PAGE: Two Dimensional polyacrylamide gel electrophoresis
FT-IR: Fourier-transform infrared spectroscopy
HPLC: High-performance liquid chromatography
MALDI: Matrix-assisted laser desorption/ionization
MS: Mass spectrophotometry
PCR: Polymerase chain reaction
RSM: Response surface method
SDS-PAGE: Sodium dodecyl sulfate-polyacrylamide gel electrophoresis
SS: *Systomus sarana*
TSA: Tryptic soy agar
