# Supplementary material for: Isolation of a bacterial strain from the gut of the fish, Systomus sarana, identification of the isolated strain, optimized production of its protease, the enzyme purification, and partial structural characterization
Source: J Genet Eng Biotechnol. 2022 Feb 10;20:24. doi: 10.1186/s43141-022-00299-3 (PMC8831710; doi:10.1186/s43141-022-00299-3)
Supplement: Supplementary file 1 — Additional file 1: Figure S1. Protease enzyme producing bacterium SS5 culture strain, Gram negative bacteria (A); PCR amplified products (B); Phylogenetic tree derived from 16S rRNA sequences after comparing with strain SS5 using neighbor-joining method (C). Figure S2. 2D-Electrophrosesis of protein extract from Bacillus thuringiensis. Table S1. HPLC analysis of protease protein extract from Bacillus thuringiensis SS5. [file 43141_2022_299_MOESM1_ESM.docx]

**SUPPLEMENTARY DATA**

**Isolation of a bacterial strain from the gut of the fish, *Systomus sarana*, identification of the isolated strain, optimized production of its protease, the enzyme purification, and partial structural characterization**

Arul Dhayalan^1^, Balasubramanian Velramar^2^, Balasubramani Govindasamy^3^, Aiswarya Dilipkumar, Perumal Pachiappan^4*^

Department of Biotechnology, School of Biosciences, Periyar University, Salem – 636011, Tamil Nadu, INDIA

………………………………………………….

**Present address:**

^1^ ICAR- National Dairy Research Institute, SRS, Adugodi, Bengaluru – 560030, Karnataka, India

^2^ Amity Institute of Biotechnology, Amity University, Raipur – 493225, Chhattisgarh, India

^3^ ICAR- Central Institute of Brackishwater Aquaculture, Chennai – 600028, Tamil Nadu, India

^4^Department of Marine Science, School of Marine Sciences, Bharathidasan University, Tiruchirappalli – 620024, Tamil Nadu, India

**Corresponding Author**:

Dr. P. Perumal

**E-mail:** perumalarticles@gmail.com

**Mobile:** +91-9443986669

**Figure S1.** Protease enzyme producing bacterium SS5 culture strain, Gram negative bacteria (A); PCR amplified products (B); Phylogenetic tree derived from 16S rRNA sequences after comparing with strain SS5 using neighbor-joining method (C).

**
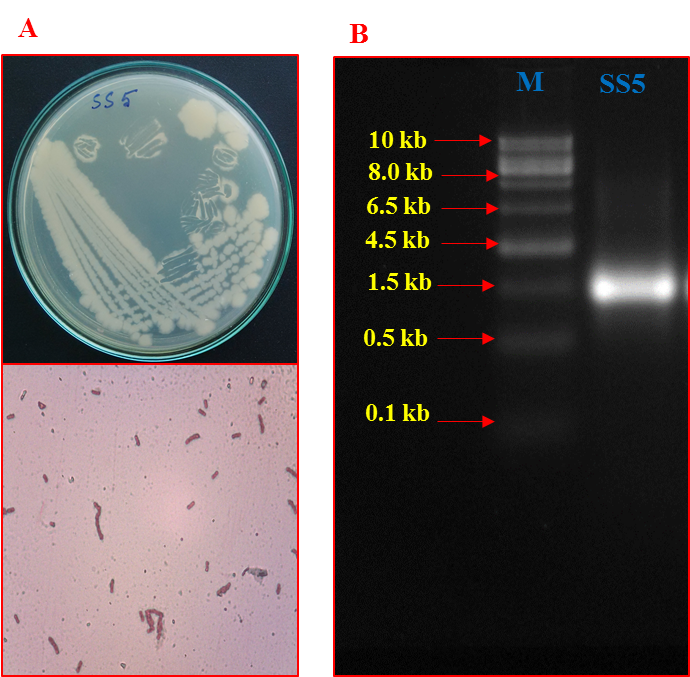
**

**
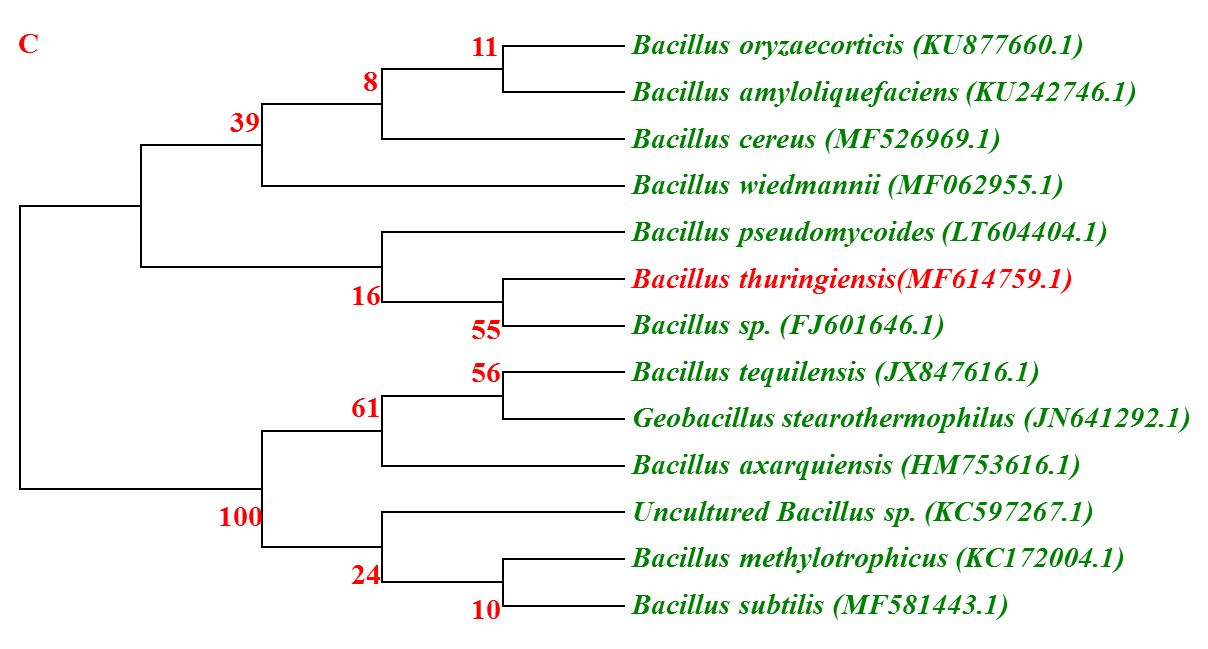
**

**Figure S2.** 2D-Electrophrosesis of protein extract from *Bacillus thuringiensis***.**

**
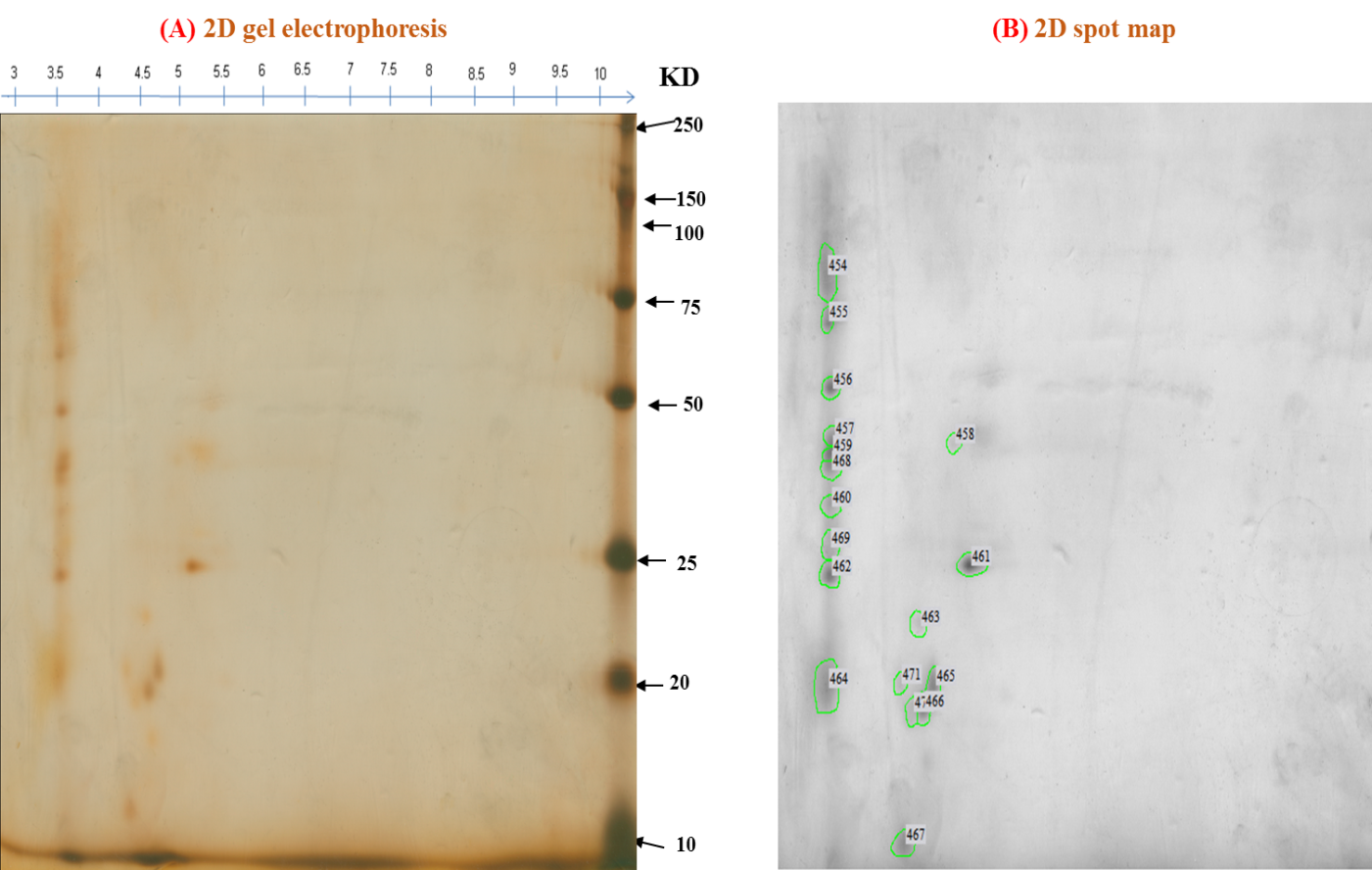
**

**Table S1. HPLC analysis of protease protein extract from *Bacillus* *thuringiensis* SS5.**

**
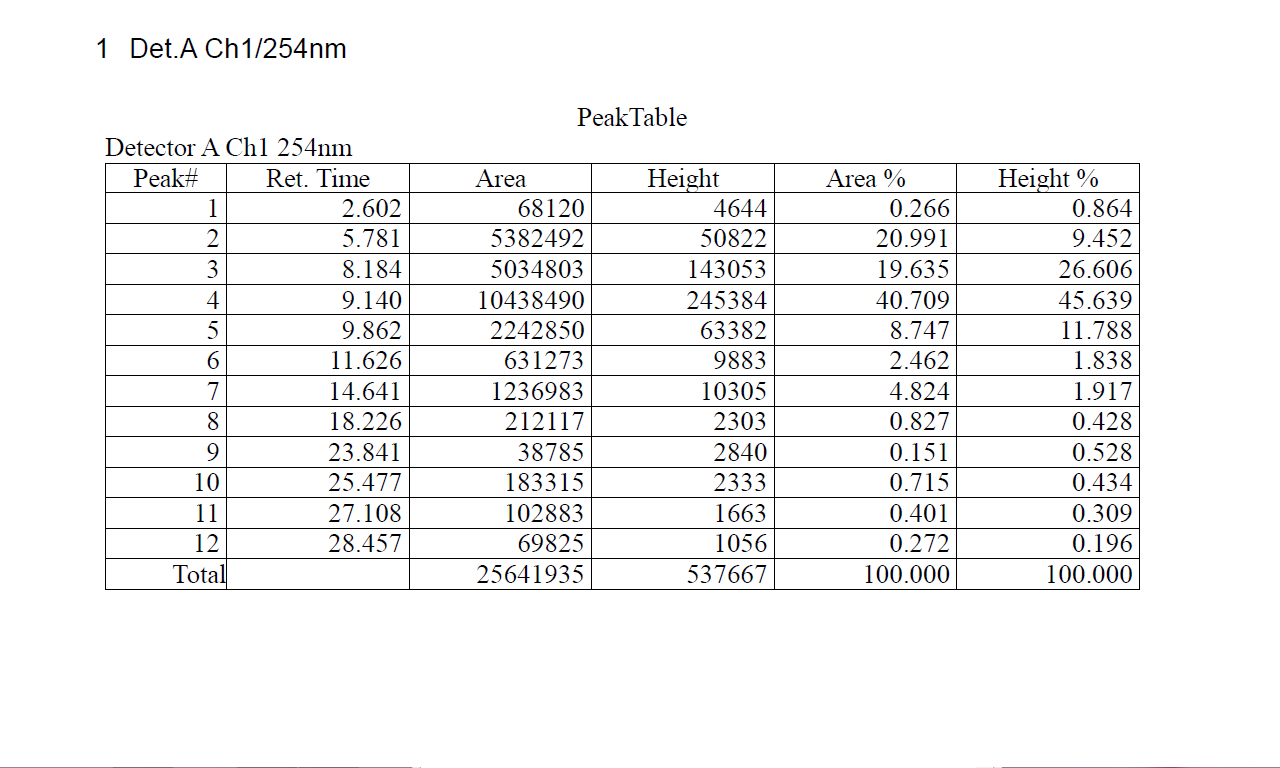
**
